# Clinical Features and Factors Contributing to Preoperative Misdiagnosis of Gastric Gastrointestinal Stromal Tumors: A Retrospective Analysis of 27 Cases

**DOI:** 10.1155/grp/6634495

**Published:** 2026-04-28

**Authors:** Jianming Xie, Jiabin Yang, Zhilong Yan

**Affiliations:** ^1^ Department of Gastrointestinal Surgery, First Affiliated Hospital of Ningbo University, Ningbo, Zhejiang, China

**Keywords:** diagnosis, endoscopic ultrasound, gastrointestinal stromal tumor, stomach misdiagnosis, submucosal tumor

## Abstract

**Objective:**

Gastrointestinal stromal tumors (GISTs) lack specific clinical manifestations and are challenging to distinguish from other gastric submucosal tumors (SMTs). This study is aimed at illustrating the atypical manifestations of non‐GISTs mimicking gastric GISTs and determining whether objective preoperative factors can help differentiate gastric GISTs from non‐GISTs in patients.

**Methods:**

We included 29 GIST patients and 27 patients preoperatively misdiagnosed with GIST located in the stomach. We compared demographic data and tumor characteristics based on endoscopic ultrasound (EUS) and computed tomography (CT) findings between GIST and non‐GIST groups.

**Results:**

Clinical symptoms in the extragastric compressions group were significantly more common (100%) than in the GISTs group (35.7%) (*p* < 0.05). Gastrointestinal stromal tumors more commonly exhibited an exophytic growth pattern than gastric non‐GISTs SMTs (*p* < 0.05). Mean arterial phase attenuation was significantly lower in gastric non‐GISTs SMTs (54.3 HU) compared with GISTs (59 HU, *p* = 0.003), with an optimal cutoff value of < 28.9 HU and an AUC of 0.689. There was a significant difference in lesion size between the GIST and gastric non‐GIST groups, with GISTs presenting larger lesions (4.2 vs. 3.3 cm, *p* = 0.038). Additionally, necrosis was more frequently observed in the GISTs.

**Conclusions:**

For gastric submucosal protuberant lesions, a comprehensive EUS and CT imaging examination is necessary for accurate diagnosis to avoid misdiagnosis and inappropriate treatment, which may affect patient prognosis.

## 1. Introduction

Gastrointestinal stromal tumors (GISTs) are mesenchymal tumors with specific histological features, primarily localized in the gastrointestinal system and abdomen [1]. GISTs most often harbor oncogenic mutations in the receptor tyrosine kinase proto‐oncogene, receptor tyrosine kinase, or platelet‐derived growth factor receptor *α* [2, 3]. The most common site for GISTs is the stomach, accounting for 50%–70% of all cases [1]. Gastric GISTs typically originate from the muscularis propria (MP) layer, leading to most patients being asymptomatic and the tumors clinically insignificant. When they occur, symptoms are nonspecific and may include abdominal pain, hemorrhage, obstruction, and intussusception [4, 5].

Due to GISTs′ lack of specific clinical manifestations, preoperative diagnosis and identification primarily rely on computed tomography (CT) examinations [6]. Confirming the diagnosis of GISTs requires pathology and immunohistochemistry examinations. The clinical manifestations of GISTs vary widely, making accurate diagnosis a challenge.

Some gastric submucosal tumors (SMTs) and lesions of extragastric compression can mimic the symptoms and endoscopic or radiological findings of gastric GISTs. Consequently, they are often misdiagnosed as GISTs preoperatively [7–9]. However, the biological behavior, prognosis, and management of these groups differ significantly. Surgical resection remains the primary and most effective treatment for GISTs [10]. In contrast, gastric SMTs other than GISTs (gastric non‐GISTs SMTs) and lesions of extragastric compression are mostly benign and tend to remain stable [11]. Some studies suggest that conservative or less‐invasive resection treatments are appropriate for benign lesions [12, 13]. Therefore, accurate preoperative evaluations that distinguish gastric GISTs from other lesions are crucial for prognostic and therapeutic decision‐making.

In this study, we illustrate various atypical manifestations of non‐GISTs that mimic gastric GISTs. Additionally, we explore whether there are objective preoperative factors that may aid in differentiating gastric GISTs from non‐GISTs in patients.

## 2. Patients and Methods

### 2.1. Patients

**Figure 1:**
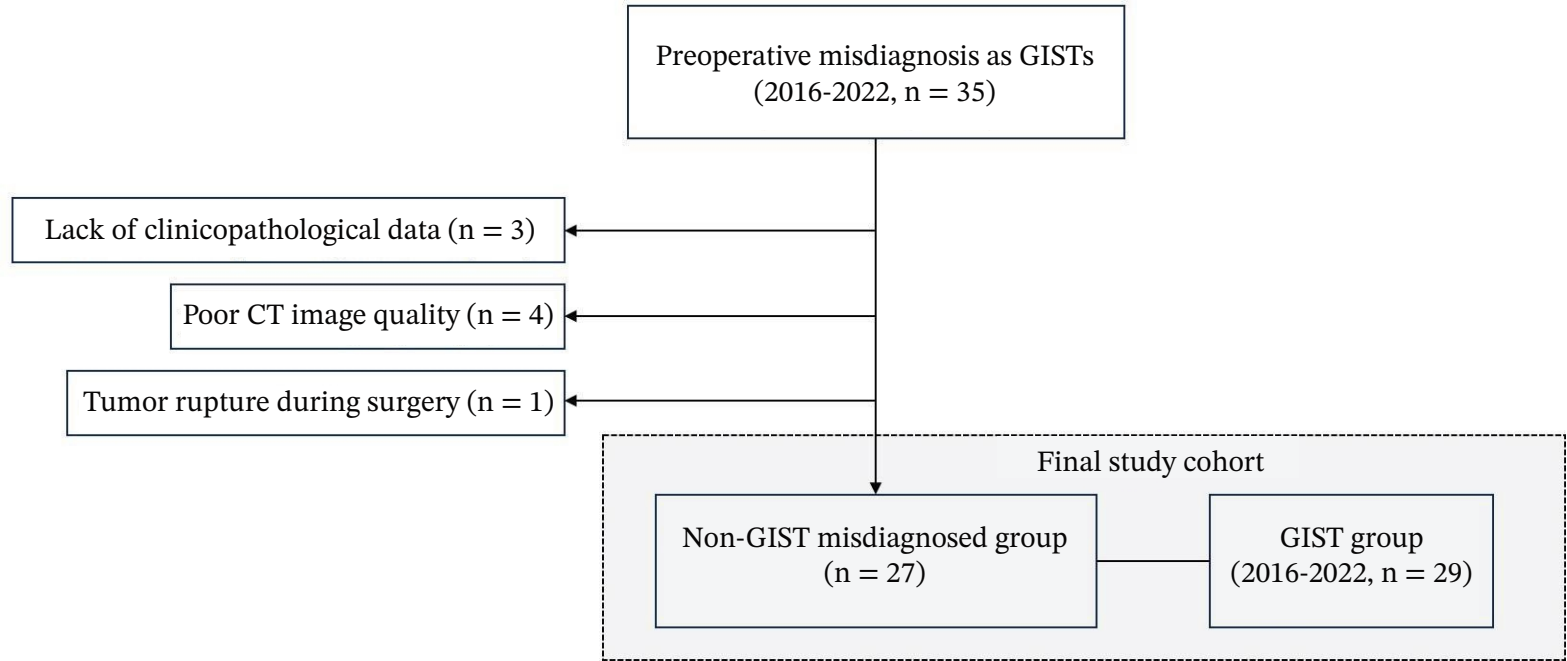
Patient enrollment and screening flowchart.

This retrospective study was approved by the Institutional Review Board of the First Affiliated Hospital of Ningbo (Ethical Approval Number: 2023049RS‐YJ01). The waiver of informed consent was granted because this study involved a retrospective review of existing medical records and imaging data, with no additional interventions or patient contact. Endoscopic findings showed submucosal elevation in both groups, and biopsy was performed under endoscopic ultrasound (EUS). Preoperative pathological biopsy confirmed stromal tumors in three patients in the stromal tumor group, and the rest were negative results. Misdiagnosed groups all had negative results. The patient screening and enrollment process is illustrated in Figure 1. Between January 2016 and December 2022, 35 patients were preoperatively misdiagnosed with GISTs in the stomach at our institution. Of these, 27 patients were included in this study based on the following criteria: (1) a preoperative misdiagnosis of gastric GIST, confirmed by EUS and CT; (2) a postoperative pathological diagnosis of gastric non‐GISTs SMTs or extragastric compression lesions following complete surgical resection; and (3) the availability of detailed clinicopathological data.

Eight patients were excluded due to the absence of detailed clinicopathological data (*n* = 3), poor image quality in CT scans (*n* = 4), and tumor rupture during surgery (*n* = 1).

Subsequently, we searched the pathological database at our institution for patients with gastric GISTs between January 2016 and December 2022. We enrolled 29 patients with GISTs in the stomach who exhibited typical clinicopathological characteristics and underwent both EUS and CT prior to surgery.

Ultimately, the study comprised 27 patients with pathologically confirmed non‐GISTs and 29 patients with pathologically confirmed GISTs.

### 2.2. EUS Examination

EUS was performed by experienced endoscopists and the model used for EUS was GF‐UCT260 (Olympus). Patients fasted for at least 6 h before the procedure. Sedation or local anesthesia was applied if necessary. The stomach was first evacuated of air using suction, and then 200–300 mL of sterile water was injected to improve acoustic coupling. The UM‐3R ultrasonic microprobe (frequency 20 MHz) was inserted through the working channel, and the lesion and surrounding tissue were scanned in multiple planes, including transverse, longitudinal, and oblique views. Necrosis under EUS was defined as areas within the lesion showing heterogeneous hypoechogenicity, loss of normal layer structure, and absence of vascular signals on Doppler imaging. The extent of necrosis was assessed qualitatively as a proportion of the lesion: mild (< 25%), moderate (25%–50%), and extensive (> 50%).

Targeted mucosal fenestration and deep excavation biopsies were then performed under EUS guidance. The obtained specimens were fixed in formalin, embedded in paraffin, and routinely stained with hematoxylin–eosin (HE). Immunohistochemical staining was performed as needed to aid in differential diagnosis. All slides were independently reviewed by two experienced pathologists, and any discrepancies were resolved by consensus to confirm the final diagnosis.

### 2.3. CT Examination

Three phase CT was performed by an experienced radiologist using GERevolution multislice spiral CT equipment. Patients were asked to fast 4–6 h before the examination and to drink 800–1000 mL of water 15 min before the examination. Patients were placed in supine position. A plain scan of the upper abdomen was first obtained. Ioversol (350 mgI/mL) was injected intravenously at 1.5 mL/kg body weight via antecubital vein at 3 mL/s. Arterial phase (AP) images were obtained at 30 s and venous phase at 60 s postinjection. Scan parameters: tube voltage 120 kV, tube current 259 mAs, slice thickness 1.25 mm, interslice distance 1.25 mm, collimator width 64 × 0.652 mm, FOV 350 × 350 mm, matrix 512 × 512. CT images were independently evaluated by two radiologists who were blinded to the pathological results. Lesion characteristics including size, growth pattern, enhancement pattern, location, AP attenuation, and presence of necrosis, calcification, ulceration, or enlarged perilesional lymph nodes (LNs) were systematically assessed. Necrosis on CT was quantitatively defined as a focal area within the lesion with attenuation values < 20 HU on contrast‐enhanced images. The proportion of necrotic area within the lesion was estimated as mild (< 25%), moderate (25%–50%), or extensive (> 50%). Any discrepancies between the two radiologists were resolved by consensus.

All patients in both groups underwent R0 resection, and two of the gastric cancer patients were confirmed to have gastric cancer by intraoperative rapid frozen pathology and additional D2 LN dissection.

### 2.4. Data Collection

Patient demographics, tumor characteristics, and follow‐up data were collected from the medical database for analysis, including (1) gender, age, and symptoms of the patients; (2) CT features, such as lesion size (in cm), growth pattern, enhancement pattern, location, AP attenuation, and presence of enlarged perilesional LNs, superficial ulcer, calcification, and necrosis; (3) EUS features including the layer of origin, echotexture, and presence of ulceration, calcification, necrosis; (4) surgical approaches; (5) detailed clinicopathological data; (6) time of misdiagnosis confirmation; and (7) follow‐up data.

Tumor growth patterns were classified as endophytic, exophytic, or mixed. The locations within the stomach were categorized as antrum, body, fundus, or cardia. The enhancement pattern was divided into heterogeneous and homogeneous. A LN exceeding 10 mm in short‐axis diameter was considered enlarged. Surface ulcerations were defined as focal lesions detected on the tumor′s surface, whereas calcifications were identified as discrete, hyperattenuating foci on noncontrast CT images. Necrosis within the lesion was determined when its CT value was below 20 HU. Morphologic assessment and immunohistochemical results postsurgical resection assisted in the pathological diagnosis.

### 2.5. Statistical Analysis

All statistical analyses were performed using IBM SPSS Statistics, Version 22.0 (IBM Corp). To ascertain differences in clinical, demographic, CT, and EUS findings between GISTs and non‐GISTs, we employed a Student′s *t*‐test or Mann–Whitney *U* test for continuous variables and chi‐square or Fisher′s exact test for categorical variables. For significant quantitative data, receiver operating characteristic (ROC) curve analysis was performed, with cutoff points calculated to maximize accuracy in differentiating gastric GISTs from non‐GISTs. The optimal cutoff value was defined as the point at which the sum of sensitivity and specificity was maximized. A *p* value of < 0.05 was considered statistically significant.

## 3. Results

### 3.1. Postoperative Pathological Analysis and Diagnosis

A total of 27 consecutive patients who were misdiagnosed with gastric GISTs before undergoing laparoscopic gastric surgery were reviewed. The final histopathology results are summarized in Table 1. Of these 27 patients, the diagnoses were as follows: five (18.5%) leiomyoma, five (18.5%) hepatic hemangioma, three (11.1%) schwannoma, two (7.4%) gastric cancer, two (7.4%) duplication cyst, two (7.4%) inflammatory myofibroblastic tumor, one (3.7%) ectopic pancreas, one (3.7%) inflammatory fibroid polyp, one (3.7%) glomus tumor, one (3.7%) neuroendocrine tumor, one (3.7%) accessory spleen, one (3.7%) metastasis SqCa (originated from gastric cancer), one (3.7%) malignant mesothelioma, and one (3.7%) lymphoepithelioma‐like gastric carcinoma.

**Table 1:** Clinical and radiologic features′ distribution between groups of GISTs and non‐GISTs.

	GISTs (*N* = 29)	Gastric non‐GISTs SMTs (*N* = 22)	Extragastric compression (*N* = 5)	*p* value GISTs versus SMTS	*p* value GISTs versus extragastric compression
Age, years (range)	60.6 (42–72)	55.3 (31–78)	58.3 (53–75)	0.174	0.627
Gender, *n* (%)				0.546	0.672
Male	13 (46.4%)	10 (45.5%)	2 (40.0%)		
Female	15 (53.6%)	12 (54.5%)	3 (60.0%)		
Location, *n* (%)				0.275	0.302
Antrum	4 (14.3%)	3 (13.6%)	0 (0.0%)		
Body	14 (50.0%)	14 (63.6%)	4 (80.0%)		
Fundus	9 (32.1%)	3 (13.6%)	1 (20.0%)		
Cardia	1 (3.6%)	2 (9.2%)	0 (0.0%)		
Clinical symptoms				0.337	0.004
Yes	10 (35.7%)	9 (40.9%)	5 (100.0%)		
NO	18 (64.4%)	13 (59.1%)	0 (0.00%)		
Growth pattern, *n* (%)				0.023	0.534
Endophytic	1 (3.5%)	6 (27.3%)	0 (0.0%)		
Exophytic	23 (82.1%)	14 (63.6%)	5 (100.0%)		
Mixed	4 (14.4%)	2 (9.1%)	0 (0.0%)		
Size, cm, mean (range)	4.2 (1.2–6.0)	3.3 (1.0–6.0)	4.4 (2.9–6.1)	0.038	0.666
AP attenuation, HU, mean (range)	59.0 (44–87)	54.3 (31–167)	44.8 (9–66)	0.003	0.276
Heterogeneous *n* (%)	21 (75.0%)	17 (77.3%)	*n* (60.0%)	0.834	0.328
Superficial ulcer, *n* (%)	7 (28.0%)	5 (22.7%)	0 (0.0%)	0.834	0.0001
Calcification, *n* (%)	0 (0.0%)	2 (9.1%)	0 (0.0%)	0.140	NA
Necrosis, *n* (%)	3 (10.7%)	0 (0.0%)	1 (20.0%)	0.270	0.559
Peri‐lesion enlarged LN, *n* (%)	0 (0.0%)	1 (4.5%)	0 (0.0%)	0.983	NA
Echotexture on EUS *n* (%)	25 (89.3%)	19 (86.4%)	3 (60.0%)	NA	0.281
Homogeneous	3 (10.7%)	3 (13.6%)	2 (40.0%)		
Heterogeneous				0.356	NA
layer of origin n (%)	5 (17.8%)	3 (13.6%)			
Second	23 (82.2%)	15 (68.2%)			
Third	0 (0%)	4 (18.2%)	5 (100%)		
Fourth					

### 3.2. Timing of Misdiagnosis Confirmation

1. Five patients with hepatic hemangioma were found to be misdiagnosed during intraoperative exploration.
2. Nine patients were identified as misdiagnosed according to intraoperative frozen section pathology. This group included one male, with diagnoses comprising two cases of duplication cyst, one glomus tumor, two gastric cancers, one neuroendocrine tumor, one inflammatory fibroid polyp, one metastasis SqCa, and one accessory spleen.
3. Postoperative histopathology confirmed misdiagnosis in 13 cases, including two inflammatory myofibroblastic tumors, one lymphoepithelioma‐like gastric carcinoma, one ectopic pancreas, three schwannomas, five leiomyomas, and one malignant mesothelioma, as shown in Table 2.

**Table 2:** The time when the misdiagnosis was confirmed.

Time of misdiagnosis confirmation	Number of cases	Misdiagnosed condition
During intraoperative exploration	5	Hepatic hemangioma
According to intraoperative freezing pathology	9	Two duplication cyst, one glomus tumor, two gastric cancer, one neuroendocrine Tumor, one inflammatory fibroid polyp, one metastasis SqCa, and one accessory spleen
Postoperative histopathology confirmed misdiagnosis	13	Two inflammatory myofibroblastic tumor, one lymphoepithelioma‐like gastric carcinoma, one ectopic pancreas, three schwannoma, five leiomyoma, and one malignant mesothelioma

### 3.3. The Final Surgical Modality

1. Gastric wedge resection (19 cases): This includes duplication cyst (two cases), glomus tumor (one case), neuroendocrine tumor (one case), inflammatory fibroid polyp (one case), accessory spleen (one case), inflammatory myofibroblastic tumor (two cases), ectopic pancreas (one case), schwannoma (three cases), leiomyoma (five cases), malignant mesothelioma (one case), and lymphoepithelioma‐like gastric carcinoma (one case).
2. Resection of hepatic hemangioma: hepatic hemangioma (five cases).
3. D2 gastrectomy: gastric cancer (two cases).
4. Total gastrectomy: gastric cancer (one case).

### 3.4. Clinical Characteristics of the Study Patients

**Figure 2:**
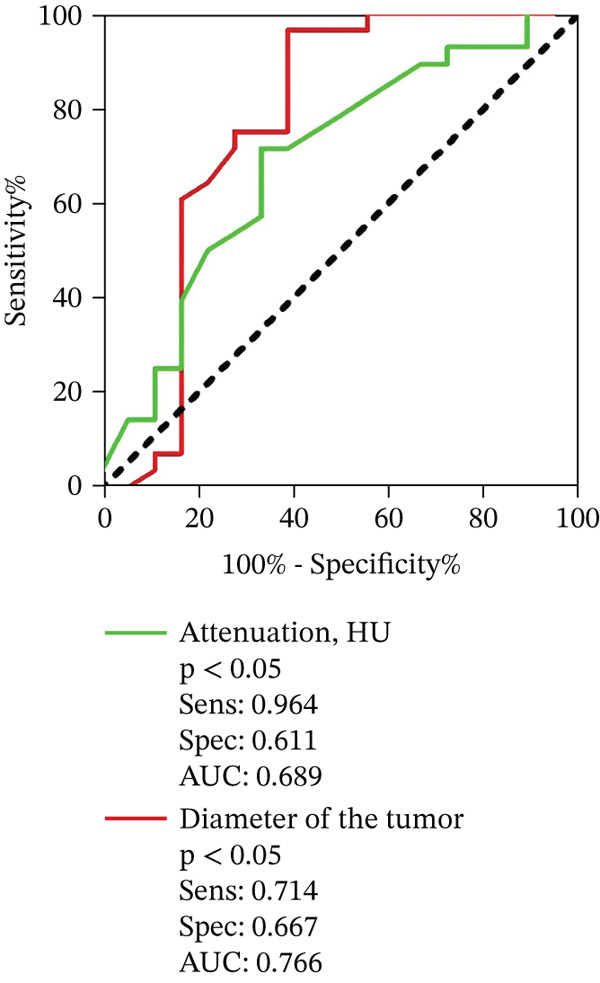
Graphs show receiver operating characteristic (ROC) curves of each reviewer for the differentiation of GISTs from gastric non‐GISTs SMTs. AP attenuation was significantly lower in gastric non‐GISTs SMTs than that in GISTs (54.3 HU vs. 59 HU, *p* = 0.003) with an optimal cutoff value of ≤ 28.9 HU and an AUC of 0.689. the median size revealed a significant difference between GISTS and gastric non‐GISTs SMTs with an optimal cutoff value of > 3.4 cm for identifying GISTs (area under the ROC curve, AUC = 0.766), and patients with GISTS were much larger than those gastric non‐GISTs SMTs (4.2 cm vs. 3.3 cm, *p* = 0.038).

**Figure 3:**
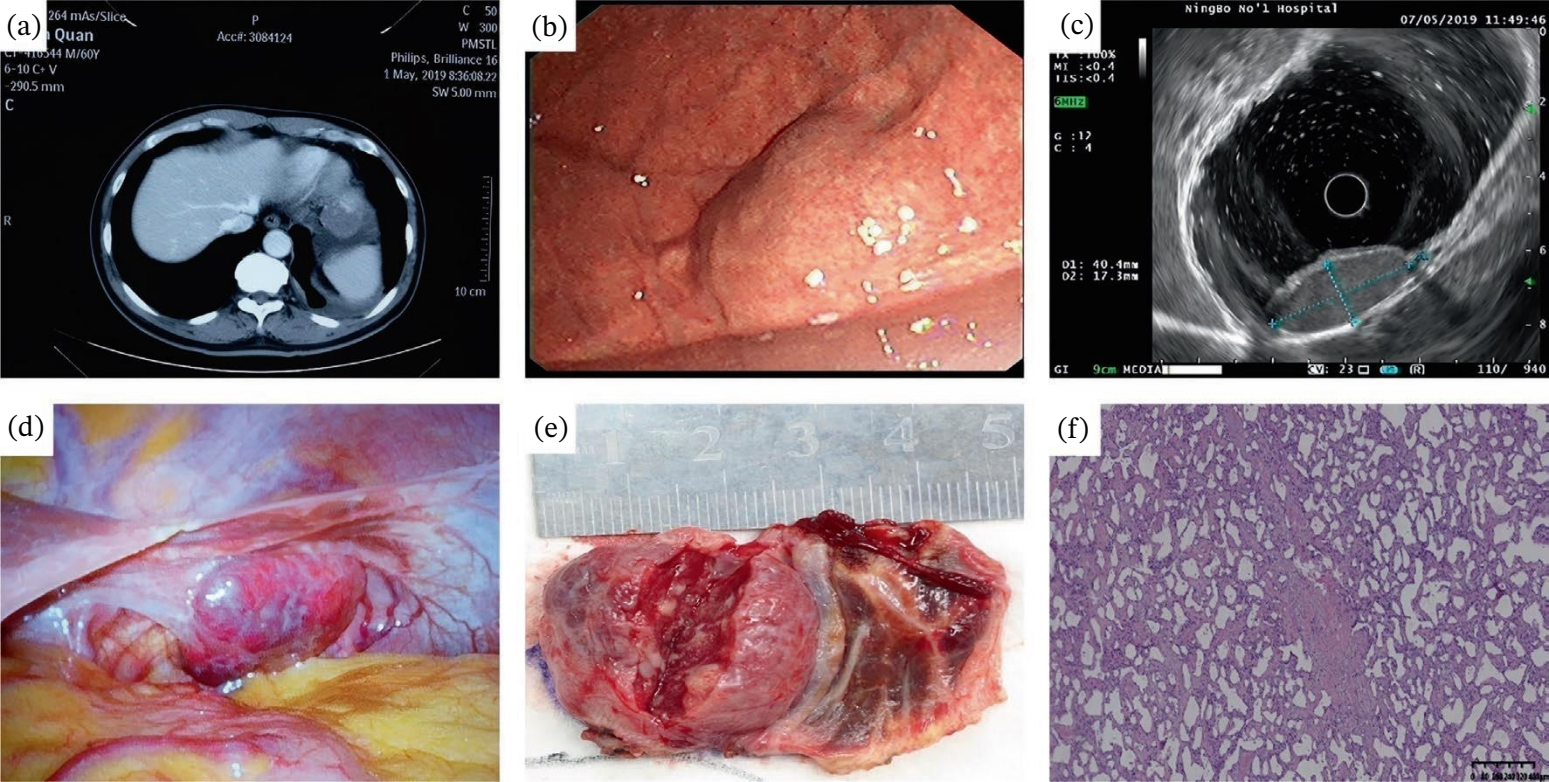
A 53‐year‐old man with hepatic hemangioma was misdiagnosed as a GIST. (a) Contrast‐enhanced computed tomography axial image showed a 2.9 cm, well‐demarcated, and outward‐protruding mass with homogeneous enhancement on the cardia. (b) Upper endoscopy showed a gastric subepithelial tumor with a size of about 3.0 cm on the cardia. (c) EUS showed a 3.0 cm well‐defined, hypoechoic with a heterogeneous echotexture, and septated lesion mainly originating in the fourth layer. (d, e) During surgery, it was confirmed that the gastric mass was a left lateral hepatic hemangioma. (f) The histopathological result of surgical resection was hepatic hemangioma (H&E, 200×).

**Figure 4:**
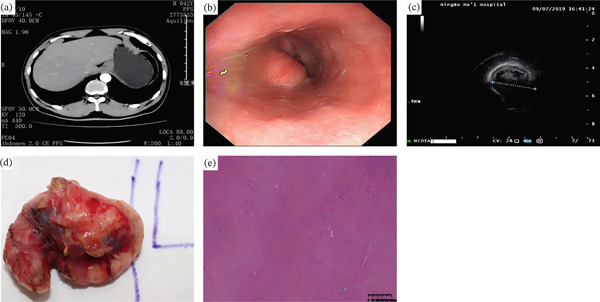
A 43‐year‐old man with gastric leiomyoma. (a) Arterial and portal phase CT images demonstrate a 2.3 cm homogeneous and low‐attenuating subepithelial mass involving the gastric cardia. (b) Upper endoscopy showed a gastric subepithelial tumor with a size of about 2.0 cm on the cardia. (c) EUS showed a hypoechoic mass in the fourth layer. (d) Gross specimen obtained after wedge resection shows an elongated subepithelial tumor. (e) The histopathological (H&E, 200×) shows the moderately cellular tumor composed of broad bundles of spindle cells.

**Figure 5:**
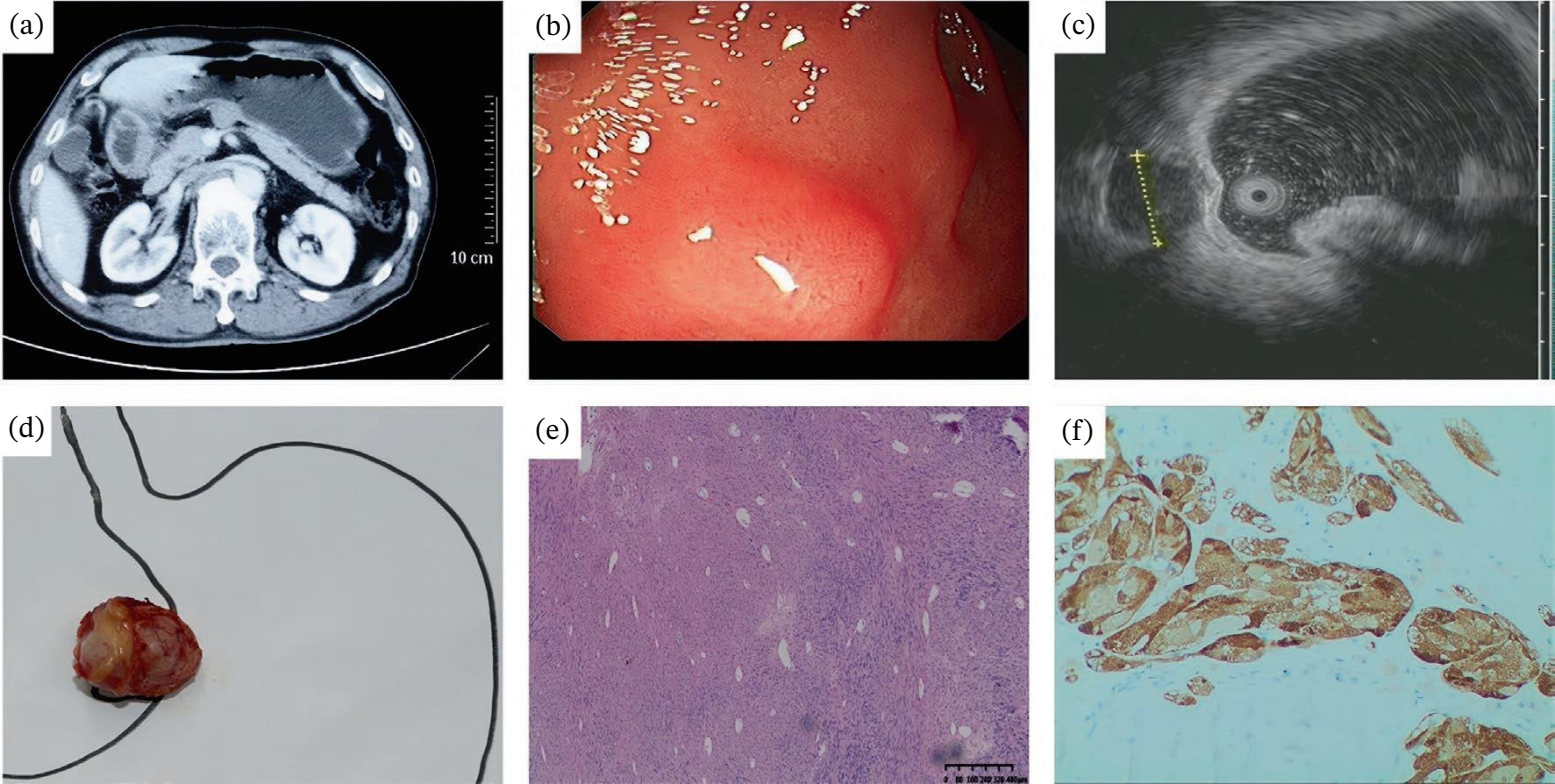
A 42‐year‐old man with gastric schwannoma. (a) A 2.0 cm homogeneous, low‐attenuating subepithelial mass was seen at the lesser curvature side of the gastric mid‐body. (b) Upper endoscopy showed a gastric subepithelial tumor with a size of about 2.0 cm on the gastric mid‐body. (c) EUS showed a hypoechoic mass in the fourth layer. (d) Gross specimen obtained after wedge resection shows a spherical type subepithelial tumor. (e, f) Tumor cells are diffusely and strongly S‐100 protein‐positive on immunohistochemistry (H&E, 200×).

**Figure 6:**
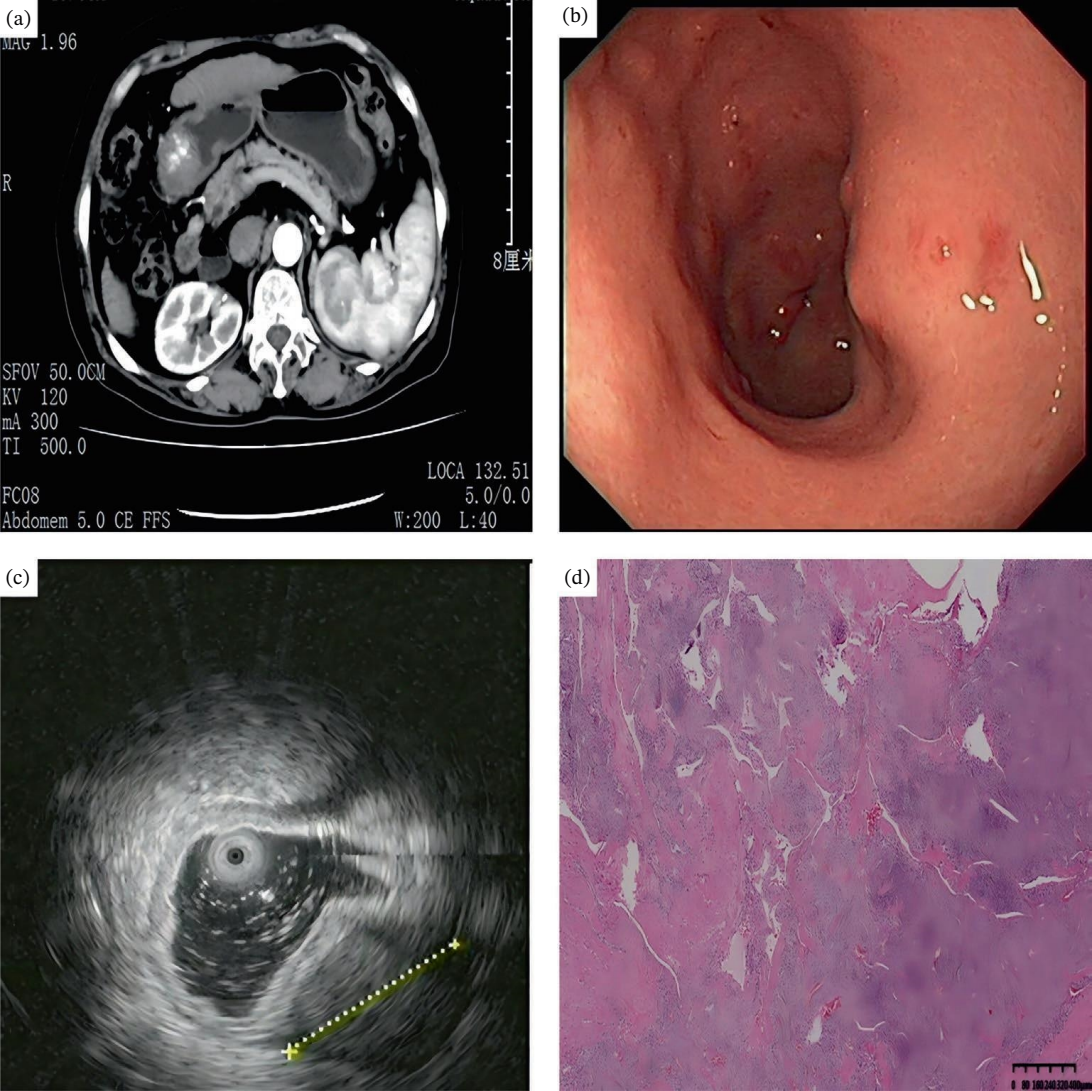
A 74‐year‐old man with gastric glomus tumor. (a) A round‐shaped mass lesion on the gastric antrum, the tumor showed nonhomogeneous enhancement; (b) esophagogastroduodenoscopy revealed a submucosal tumor on the greater curvature of the antrum with bridging fold; (c) endoscopic ultrasonography showed that the tumor was located on the muscle layer; (d) proliferating oval‐shaped cells in a small nest formation and a high nuclear cytoplasmic ratio were observed (H&E, 200×).

**Figure 7:**
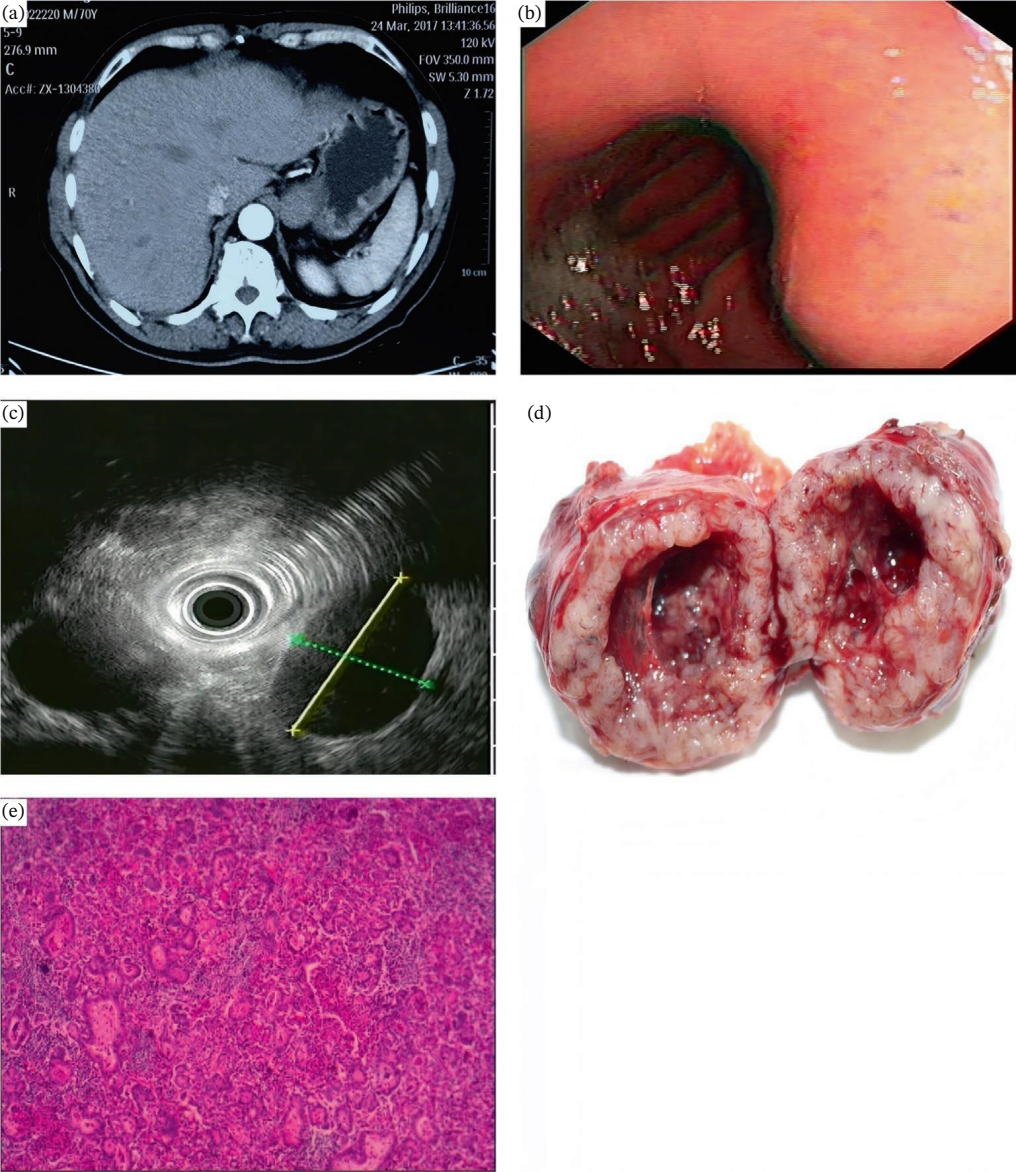
A 70‐year‐old man with lymphoepithelioma‐like gastric carcinoma. (a) Tumor in the cardia observed by abdominal computed tomography scan was enhanced; (b,c) a 3.0 cm, round, nonulcerated lesion in the lesser curvature of the cardia, as revealed by esophagogastroduodenoscopy; (d) gross specimen obtained after wedge resection showed a spherical type subepithelial tumor; (e) dense lymphocytic infiltration of the tumor stroma, revealed by hematoxylin and eosin staining of the postoperative specimen (H&E, 200×).

The clinical and radiologic characteristics of 56 patients, including 29 cases with GISTs, five cases with extragastric compressions, and 22 cases with gastric non‐GISTs SMTs, are summarized in Table 1. The gender distribution and mean age showed no significant differences among the three disease groups (*p* > 0.05). Clinical symptoms were significantly more common in the extragastric compressions group (5/5, 100%) compared with the GISTs group (10/29, 35.7%) (*p* = 0.004). However, there was no significant difference between the gastric non‐GISTs SMTs group (9/22, 40.9%) and the GISTs group (*p* = 0.337). GISTs commonly exhibited an exophytic growth pattern more frequently than gastric non‐GISTs SMTs (82.1% vs. 63.6%). The mean AP attenuation was significantly lower in gastric non‐GISTs SMTs compared with GISTs (54.3 HU vs. 59 HU, *p* = 0.003), with an optimal cutoff value of < 28.9 HU and an AUC of 0.689 (Figure 2). The lesion size differed significantly between GISTs and gastric non‐GISTs SMTs, with an optimal cutoff value of > 3.4 cm for identifying GISTs (AUC = 0.766). Patients with GISTs had larger lesions than those with gastric non‐GISTs SMTs (4.2 vs. 3.3 cm, *p* = 0.038) (Figure 2). Additionally, necrosis was observed more frequently in the GISTs. No statistically significant differences were found in the irregular margin, echotexture on EUS, and perilesion enlarged LN (*p* > 0.05) among the three disease groups (Table 1). Representative examples are presented in Figures 3, 4, 5, 6 and 7.

### 3.5. Follow‐Up Data

Of the 27 patients who were preoperatively misdiagnosed as having GISTs, follow‐up was conducted over a median period of 46 months. One patient passed away due to systemic metastasis 12 months postprocedure. The remaining 26 patients were alive and showed no evidence of tumor recurrence or metastasis.

## 4. Discussion

GISTs, originating from the interstitial cells of Cajal or their precursors, represent a group of mesenchymal neoplasms with varying malignancy potentials [14]. SMTs arising in the MP layer are usually asymptomatic, necessitating differentiation between gastric GISTs and other clinically benign gastric SMTs from the same layer [15, 16]. The histopathological types of SMTs are diverse, encompassing benign lesions like leiomyoma, schwannoma, lipoma, and ectopic pancreas, as well as potentially malignant tumors such as GIST, leiomyosarcoma, and neuroendocrine tumors [17]. The clinical and imaging manifestations of GISTs lack specificity, and many clinicians, focusing too heavily on single examination results for diagnosis, often overlook clinical symptoms. This leads to frequent misdiagnoses or overtreatment. In this study, we reviewed recent misdiagnosed cases of gastric GIST and found that 27 were erroneously diagnosed as GIST, including 18 other gastric SMTs, three gastric cancers, one lymphoepithelioma‐like gastric carcinoma, and five extragastric compression lesions, all of which were left hepatic hemangiomas. All misdiagnosed gastric SMT cases were confirmed as such by pathology after local wedge gastrectomy.

Given the submucosal origin of GISTs, acquiring pathology information via routine endoscopic biopsy before surgery is challenging. Most clinical units base their diagnosis and treatment decisions on EUS or CT findings combined with personal experience. Preoperative diagnosis of gastric SMTs can be aided by CT scans, which offer several advantages. Choi et al. [7] used heterogeneous enhanced CT to differentiate large gastric GISTs from SMTs, achieving a diagnostic accuracy of 87%–90%. They noted that characteristics such as larger lesion size, noncardial location, presence of necrosis, heterogeneous enhancement, and absence of lymphadenopathy are indicative of large GISTs when differentiating from leiomyomas or schwannomas. In this study, the mean AP attenuation of gastric non‐GIST SMTs was lower than that of GISTs. The tumor size in GIST patients tended to be larger than in those with gastric non‐GIST SMTs. Although a threshold of > 3.4 cm may provide reference information, it should not be considered a definitive diagnostic criterion. Research on large datasets indicates that GISTs typically exhibit larger tumor sizes [18, 19]. This discrepancy may be attributed to the benign nature and slower growth of gastric non‐GIST SMTs [20]. Accounting for the majority of intramural tumors, GISTs can present variably, ranging from small intraluminal lesions to large exophytic masses protruding into the peritoneal cavity, often with areas of hemorrhage or necrosis [21].

Being adjacent to the liver, colon, pancreas, and spleen, the stomach is susceptible to extragastric compression. Lesions from these adjacent organs can compress the gastric wall, leading to misleading images and false signs of submucosal eminence during endoscopic observation, particularly when the stomach is inflated [22]. In our study, we identified five cases of extraluminal compression caused by left hepatic hemangioma. Such compression of the stomach by neighboring diseased organs can easily lead to misdiagnosis if reliance is placed solely on endoscopy. This can result in unnecessary blind surgery or endoscopic treatment.

EUS biopsy is an important preoperative diagnostic tool, which provides physicians with a wealth of lesion information, helps to establish an accurate preoperative diagnosis, guides the choice of treatment options, and assesses the necessity and risk of surgery [23–25]. Okasha et al. [23] evaluated the accuracy of EUS and endoscopic ultrasound‐guided fine needle aspiration (EUS‐FNA) in the diagnosis of endoscopic biopsy‐negative GI tumors and showed that EUS combined with EUS‐FNA is an accurate procedure for the diagnosis of endoscopic biopsy‐negative GI tumors. Furthermore, although ultrasound applications, including EUS and IDUS, are valuable, they should not be the only diagnostic criteria used. EUS can accurately determine the originating layer of subepithelial tumors within the gastric wall. In contrast, CT primarily assists in determining the growth pattern of tumors. Therefore, a combination of CT and MRI, based on EUS findings, is a rational approach for most GIST diagnoses and must be emphasized. CEUS is a newer modality than contrast‐enhanced CT and significantly improves the detection of blood flow signals using contrast microbubbles. CEUS has attracted increasing attention for its convenience, lack of radioactivity and risk of nephrotoxicity, and real‐time observation of dynamic perfusion of lesions [26–28]. Fang et al. [26] found that CEUS has a high diagnostic value for hypoechoic hepatic hemangiomas with peripheral nodular or annular enhancement, concentric filling, “fast‐in and slow‐out” and “slow‐in and slow‐out” phase transitions. Crinò et al. [29] reported that the hypoenhancement pattern of CEUS schwannomas may help to achieve diagnosis and during FNA, solid components must be targeted, and EUS‐FNA allows appropriate preoperative differential diagnosis.

Overall, although CT, EUS, and CEUS can provide helpful information, these imaging features should be interpreted in conjunction with clinical findings and other diagnostic modalities and should be considered as supportive rather than definitive for diagnosis.

Additionally, recent studies in locally advanced rectal cancer underscore the utility of multimodal preoperative evaluation and predictive modeling for optimizing therapeutic response. The NECTAR trial [30] and SPEED study [31] demonstrated that integrating neoadjuvant chemoradiotherapy with PD‐1 blockade yielded favorable pathological complete response rates, and that incorporating additional predictive variables, such as baseline gut microbiome characteristics, significantly enhanced patient stratification. Moreover, the NCRT‐PD1‐LARC trial [32] highlights that a well‐designed, multicenter, prospective framework enables rigorous assessment of both safety and efficacy. Collectively, these findings support the concept that the integration of complementary diagnostic and predictive modalities including imaging, clinical parameters, and molecular biomarkers can substantially refine preoperative decision‐making. By analogy, similar principles apply to gastric submucosal lesions. Recent studies have shown that integrating imaging features with clinical and laboratory data improves preoperative assessment: gastric GISTs from different locations exhibit distinct clinical and prognostic profiles [33], preoperative albumin (ALB), CEA, tumor location, and Ki‐67 expression predict LN metastasis in gastric neuroendocrine tumors [34], and changes in serum ALB levels independently predict short‐term postoperative complications after gastrectomy [35]. Collectively, these findings support a combinatorial approach incorporating EUS, CT, CEUS, and relevant clinical and laboratory features to improve diagnostic precision and optimize patient management.

Gastric cancer, originating from mucosal epithelium, can typically be confirmed pathologically via a gastroscope before surgery [36]. However, if a small number of mass gastric cancers fail to yield definitive pathology through biopsy, they may be misdiagnosed as GISTs. Such misdiagnoses can lead to fundamental errors in the surgical approach. In our study, one patient with gastric antrum cancer was initially misdiagnosed as GIST and did not undergo timely frozen pathology for differential diagnosis, resulting in a subsequent additional operation for a D2 distal gastrectomy. It is crucial to note that the pathological diagnosis from endoscopic gastric biopsies differs significantly from surgical biopsies of gastric cancer, and these differences should be considered in the diagnostic process. Emphasizing a preoperative comprehensive evaluation, including endoscopy, CT, serum tumor markers, and family genetic history, is necessary to avoid incorrect surgical decisions.

This study has several limitations. First, because only 27 preoperatively misdiagnosed cases were included, the present work should be regarded as exploratory. The small sample size inevitably reduces the robustness of the statistical estimates, and therefore the findings must be interpreted with caution. Although some imaging features reached statistical significance, these results are better understood as preliminary observations rather than definitive diagnostic criteria. Second, although effective indicators to distinguish gastric stromal tumors from nonstromal lesions are likely multifactorial, our study focused on readily available imaging features, primarily lesion size and AP attenuation. Other clinical variables, such as symptom duration and tumor markers, were not consistently recorded and therefore could not be included. Finally, future prospective, multicenter studies are warranted to collect more comprehensive clinical and molecular data, which could further improve the diagnostic discrimination of gastric submucosal lesions.

## 5. Conclusion

In this study, we retrospectively analyzed the EUS and CT image data of 27 patients who were preoperatively misdiagnosed as GISTs. GISTs showed an exophytic growth pattern more often than SMTs, with larger tumor sizes and higher AP CT attenuation values. Follow‐up data suggest that accurate diagnosis is essential for making treatment plans and assessing patient outcomes.

**Nomenclature**
AP: arterial phase
CEUS: contrast‐enhanced ultrasound
CT: computed tomography
EUS: endoscopic ultrasound
EUS‐FNA: endoscopic ultrasound‐guided fine needle aspiration
GIST: gastrointestinal stromal tumor
HE: hematoxylin–eosin
IDUS: intraductal ultrasound
LN: lymph node
MP: muscularis propria
ROC: receiver operating characteristic
SMT: submucosal tumor
SqCa: squamous cell carcinoma

## Author Contributions

Jianming Xie equal contribution conceived the study and Jiabin Yang participated in its design and statistics, and Zhilong Yan helped in drafting the manuscript.

## Funding

This study was supported by the Medical Science and Technology Project of Zhejiang Province (10.13039/501100017594, 2020KY813). Medical Science and Technology Project of Zhejiang Province (2025KY249).

## Disclosure

All authors read and approved the final manuscript.

## Ethics Statement

This retrospective study was approved by the Institutional Review Board of the First Affiliated Hospital of Ningbo (Ethical Approval Number: 2023049RS‐YJ01). The waiver of informed consent was granted because this study involved a retrospective review of existing medical records and imaging data, with no additional interventions or patient contact.

## Consent

The authors have nothing to report.

## Conflicts of Interest

The authors declare no conflicts of interest.

## Data Availability

The data that support the findings of this study are available from the corresponding author upon reasonable request.
